# What Does the Body Communicate With Postural Oscillations? A Clinical Investigation Hypothesis

**DOI:** 10.3389/fpsyg.2021.668192

**Published:** 2021-06-16

**Authors:** Andrea Buscemi, Santi Scirè Campisi, Giulia Frazzetto, Jessica Petriliggieri, Simona Martino, Pierluca Ambramo, Alessandro Rapisarda, Nelson Mauro Maldonato, Donatella Di Corrado, Marinella Coco

**Affiliations:** ^1^Department of Research, Italian Center Studies of Osteopathy, Catania, Italy; ^2^Department of Neuroscience and Reproductive and Odontostomatological Sciences, University of Naples Federico II, Naples, Italy; ^3^Department of Human and Social Sciences, Kore University, Enna, Italy; ^4^Department of Biomedical and Biotechnological Sciences, University of Catania, Catania, Italy

**Keywords:** body communication, postural oscillations, osteopathic medicine, health, upright posture

## Abstract

The evolution of the foot and the attainment of the bipedia represent a distinctive characteristic of the human species. The force of gravity is dissipated through the tibial astragalic joints, and the movement of the ankle is manifested on a sagittal plane. However, this is in contrast with other studies that analyze the straight station in bipodalic support of the body. According to these studies, the oscillations of the body dissipated by the articulation of the ankle are greater on a frontal plane than on a sagittal plane. Probably, this can be deduced by analyzing the concept of “cone of economy (COE) and equilibrium;” a cone that has its base with the oscillations described by the 360° movement performed by the head and has its apex that supports polygon defined by the tibio-astragalic articulation. The purpose of this study was to evaluate a kind of communication between the oscillations of the COE and equilibrium and the main sphere of somatic dysfunction (structural, visceral, or cranial sacral), assessing the reliability of the “fascial compression test.” The implications of this connection have been considered, while grounding the hypothesis in the ability of the human body to maintain its center of mass (COM) with minimum energy expenditure and with minimum postural influence. At the same time, the fascial compression test provides a dominant direction of fascial compartments in restriction of mobility.

## Introduction

In the osteopathic medicine field, therapeutics and clinical goals are focused on findings and treatments of somatic dysfunctions, which is described as an impaired or altered function of related components of the somatic (body framework) system, caused or expressed by any change of biological tissue structures in size, texture, structure, and position. However, until recently, the uncertainty linked to the pathophysiological processes leading to the onset of dysfunction and the lack of diagnostic reliability, based on tissue indicators, such as abnormalities in the cellular texture, asymmetries, restrictions of mobility, and greater tenderness or sensitivity, make the concept of “osteopathic lesion” a subject of debate and further research (Liem, 2016). Hence, this study aimed to propose and develop a method for assessing the dysfunctional sphere, using knowledge of the communication points between the fascial system and the oscillatory adaptive capacity of the body in space to maintain an upright posture.

The physiology behind the upright posture in the human could appear as a static, stable, and imperturbable condition. The truth, however, seems to be much more complex and is still debated nowadays (Pollock et al., 2000). There is not yet a standard and universal model capable of correctly understanding and recognizing all the variables behind the physiology of postural control in humans.

For most adult individuals, the bipedal stance is a very simple and automatized task; however, the physiological processes inherent to this condition are quite complex and finalized to maintain and balance the body mass. This can be intrinsically unstable due to the gravitational load and the continuous perturbations caused by internal stimuli, such as heart beating and breathing, or external stimuli, including visual distortions or changes in the environment surrounding the individual (Forbes et al., 2018).

In order to maintain a stable balance in an unstable body, it is important to keep correct coordination and communication between the sensorineural system and musculoskeletal system, aiming to maintain the center of mass (COM) of the body inside the base of support (BoS) edges. This physiological function requires an unavoidable and necessary shift of the entire COM in a horizontal plane (Haddas and Lieberman, 2018). These continuous changes in the COM are expressed in the form of oscillations of the entire body, which, in physiological conditions, occur inside a stable region of the space surrounding the individual. This is called the cone of economy (COE; Dubousset, 1994), and it has a conical shape with the apex facing the BoS and the base facing the head. Any deviation and shifting from the center of the cone represent a challenge to keeping balance, requiring more muscle activation and energy expenditure (Savage and Patel, 2014).

Therefore, oscillations inside the COE represent individual dynamic skills in order to compensate perturbations caused by internal and external stimuli to maintain all those mechanical and physiological processes useful for maintaining balance and therefore the upright posture function.

The concept of stability and balance in human biomechanics is not well defined and universally recognized yet. However, it is possible to roughly adapt Newtonian mechanics principles to the biomechanical principles related to human balance physiology and the concepts behind the COE. In mechanical physics, the term “balance” is defined as the state of an object when the resultant load actions (forces or moments) acting upon it are zero (Newton’s First Law). In the case of unstable bodies, the more the line of gravity passing through the COM is distant from the center of BoS, the more precarious the balance will be. In inanimate objects, physical laws enable the fall of the object to be taken into consideration, if the line of gravity falls outside the BoS. The sensorineural and muscular postural control of the human body, on the other hand, guarantees muscle activation forces against gravitational forces to ensure the maintenance of COM within the BoS (Pollock et al., 2000). These principles are reflected in the definition of COE given by Dubousset (1994) and in the concept of postural alignment, or ideal posture, which is defined as that state in which body mass is distributed in such a way that muscles tone, bones, and ligaments neutralize the force of gravity (Kappler, 1982).

In orthopedic surgery, postural models describe upright posture physiology as a complex relationship between the spine and pelvic morphology with axial and appendicular musculature. Their ideal alignment allows for the maintenance of a bipedal state with minimum energy expenditure (Savage and Patel, 2014). However, these models find it hard to consider important factors involved in upright postures, including the interdependence of body systems relative to a somatic framework, fascial mechanics, and bio-psycho-social dynamics of the individual (Lunghi et al., 2016).

In the osteopathic field, the “ideal posture” concept was defined by Kuchera (1995) as the result of dynamic interactions between individual force and gravity force. Posture, therefore, becomes an expression of the balance between these two forces and thus, an indicator of the adaptive capacity of an individual against the force of gravity. A recent review of the biomechanical model, defined as the integration between somatic components relative to the upright posture function, suggested that the “ideal posture” is a temporary and constantly dynamic result based on individual capacities expressed and integrated at a neuro-myofascial level and based on the interdependence of neural, somatic, and biopsychosocial systems (Lunghi et al., 2016).

## Hypotheses

This study hypothesis aimed to analyze structural and organics elements involved in bipedal stance. They attempt to assess and possibly add to the equation the mechanics and the nature of the fascial system. Thinking about the concepts expressed by the COE and its relationships with the physiological mechanisms of posture, it would be appropriate to ask: Does a deal posture correspond to an ideal oscillatory sequence?

In the stance phase, body mass oscillations occur through fixed supports represented by the ankle and hip joints. Adaptations in body sways can occur on a sagittal plane, with anteroposterior oscillations, or on a frontal plane with mediolateral standing balance. During unperturbed postural conditions, the ideal oscillation patterns (ergonomic and with minimum energy expenditure) occur on a sagittal plane, with body sways equally distributed in an anteroposterior way (Forbes et al., 2018). The bone anatomy of the ankle joint, the relationships between the head of the talus and the tibiotarsal mortar and related soft tissue mechanics, seems to confirm to this predictive model. Only the geometry of this joint, together with a review of its biomechanics, confirms that, in the bipedal stance, range of movement is induced and facilitated primarily on a sagittal plane, providing resistance to eversion (Brockett and Chapman, 2016).

On the other hand, the knowledge of the authors about mediolateral standing balance in the frontal plane is much less clear and defined, as this also involves load/unload mechanisms by the hip abductors and adductors with a minimum counterbalancing activity from ankle invertors and evertors (Forbes et al., 2018).

This study aimed to assess individual capacity to discharge gravitational forces through body oscillations inside the COE, using the functional assessment of the body system based on the specificity of their structure. These functional evaluations should define “non-ideal” oscillations patterns, revealing postural perturbations showing a possible dysfunctional sphere. This leads the operator to follow a determined clinical rationale based on the knowledge and a treatment relative to the main body systems in the body, which include the musculoskeletal system, the visceral system, and the cranial system with their respective and specific techniques and treatment modalities. Besides internal and external stimuli cited before, further postural perturbation factors, acting upon body sways, could reside in the fascial system and its mechanics.

## The Role of the Fascial System in Maintaining an Upright Posture

Several studies have shown the important role of the fascial system in controlling and maintaining an upright posture. Inherent myofascial tissue properties seem to play a decisive passive role, due to the tissue stiffness itself, in bipedal stance together with the active action of the neuromuscular and sensory systems. Therefore, these myofascial physiological properties occur without the control of the central nervous system. Indeed, as for the structure and histological anatomy of the myofascial tissue, it presents “tensegritive” properties capable to absorb and transmit gravity forces, generating a framework capable of supporting body mass and drastically reducing the neuromuscular effort required to maintain COM within the BoS. This inherent myofascial tissue property is called “Human Resting Myofascial Tone” and is integrated with the other passive body support systems, such as ligaments and bones (Masi et al., 2010).

The fascial system as connective tissue is rich in collagen fibers. It has been observed that the structural disposition of these fibers follows the main compression pathways mechanically defined when the entire body mass is under compressive forces, such as the force of gravity. Indeed, looking at the functional hierarchies of myofascial tissue, the epimysium presents bundles of collagen type I fibers disposed of longitudinally with respect to the sarcomere, forming a helical pattern arrangement at 55° relative to the longitudinal resting fusiform muscles axis. This angle is ideal for absorbing and transmitting both tensive and compressive forces (Scarr, 2016).

For example, lumbar multifidus has an inherent stiffness capable of stabilizing and maintaining an upright posture. Therefore, human resting myofascial tone can be considered a passive function of the myofascial tissue, due to its elastic and stiffness properties. These specific properties occur only in a stationary phase, as viscoelastic properties of the fascial tissue tend to change during active movements. In short, during postural control, the fascia acts as a spring, without hysteresis, and is therefore useful for managing adaptive oscillations expressed inside the COE. This is a further confirmation of how the fascial system plays a decisive role in balancing and supporting an upright posture (Masi et al., 2010; Francavilla et al., 2020).

However, it can also be a source of perturbation and an additional force that acts on balancing body mass. Therefore, it is a variable to be added within the interceptive stimuli that act on postural control (Tozzi, 2015).

The fascial influence on posture has already been studied with significant benefits on postural assessment, followed by specific lengthening protocols of the main myofascial meridians (DellaGrotte et al., 2008).

Furthermore, there seems to be a possible communication between hypertonicity of the axial fascia and the onset of ankylosing spondylitis (Masi et al., 2010). Some fascia-related mechanisms seem to be associated with the somatic dysfunction model. For example, fascial ability to change its structure and texture-based upon environmental demands can increase stiffness and surrounding tensional states in soft tissues, with loss of elastic properties and the consequent inability of the tissues to properly absorb or transmit forces, such as gravity force (Tozzi, 2015). In view of these considerations, it seems reasonable to hypothesize that the COE should express higher amplitude oscillations toward the region of the human body with the most probable presence of somatic dysfunction. This phenomenon should occur as the result of the altered tensional state of the specific tissues, i.e., a passive, constant, and low-frequency postural perturbation disconnected from the central nervous system, but can easily be compensated through neuromuscular activations during postural control in an upright posture.

Curiously, similar anti-gravity mechanical properties have been observed not only in myofascial tissue related to the muscle skeletal system but also in connective tissue, which composes and gives shape and function to the visceral fascia and meningeal fascia (Nakagawa et al., 1994; Maikos et al., 2008; Stecco et al., 2017). Considering the evidence behind these tissue properties and the anatomical dispositions of the main fascial systems, evaluating dysfunctional oscillations patterns through a manual assessment of the COE is possible, guiding the manual therapist first toward a clinical approach, based on the proper rationale and techniques.

## Evaluation of the Hypothesis

On summarizing, fascia is a ubiquitous viscoelastic tissue that forms a functional collagen matrix that surrounds and permeates all the body structures (somatic, visceral, neural, and vascular). This has made it much difficult, during years of research, to name and histologically isolate it. Many researchers have tried to define various types and nomenclatures of fascial tissues based on the relationship between their structures and their functions; therefore, fascial nomenclature has been studied and debated. In order to assess the influence of the somatic dysfunction inside the COE, it is essential to know the relationship between the main fascial systems and their anatomical disposition around the human body framework.

The functional viscoelastic model, inherent to the myofascial tissue, is studied and described by Masi et al. (2010). It can be useful in a first partition and nomenclature of fascia. As an important anti-gravity and postural component, it is abundantly present in the body region posterior to the spine. As mentioned above, the lumbar multifidus is a perfect example.

The histological model described by Kumka and Bonar (2012) highlighted another functional fascial definition of clinical relevance. For example, the so-called “passive linking fasciae” seem to have a decisive role in passive force transmission. The latest evidence in research shows a specific fascial functioning with its histological mechanics, relative to that part of the somatic framework responsible for the upright posture function. On the other hand, the “separating fascia” system has the main function of compartmentalizing organs and body regions and maintaining their structural and functional integrity by promoting a correct sliding between viscera during movements and a good absorption and adaptation to mechanical stresses, as well as limiting the spread of infections (Liem, 2016).

The role of the latter anatomical structures on the maintenance of an upright posture is unclear and poorly studied. However, there are some histological and biomechanical considerations to take into account. The protective and compartmental function of the meningeal system on the central nervous system and spinal cord is universally recognized. It is also known that the human spine is stabilized not only by musculoskeletal components but also by soft tissues, through the fascia and spinal ligaments, both in the dynamic phase and in the stationary phase. Within these tissues, posterior longitudinal ligament (PLL) and spinal dura mater (SDM) are present. Evidence from histological examinations has outlined how the abundant presence of collagen and elastin fibers, arranged in a caudal sense, both in the PPL and in the ventral portion of the SDM, is extremely important in longitudinal mechanical stress responses. Furthermore, they provide resistance to tensions caused by the flexion of the spine, preventing SDM folding during hyperextension (Nakagawa et al., 1994). Also, the inherent connective tissue stiffness seems to play an important role in system stability.

Other histological research showed how SDM longitudinal fibers in the lumbar spine are 5–10 times denser than transversal fibers (Maikos et al., 2008). It seems like some of the functions inherent to soft tissue mechanics are linking, transferring, maintaining, and discharging longitudinal forces. Changes in SDM tensional states are necessary to alleviate postural stress and flexion/extension movements. Dural tissue acts in the same way as the collagen fascial tissues, through fibroblasts action, altering the surrounding textures.

The suboccipital region hosts an important anatomical component that connects the somatic framework with the dural system. It is called myodural bridge (MDB), and it is composed of fibrous connective tissue between the dura mater and the following:

1. Rectus capitis posterior minor (RCPmi).
2. Rectus capitis posterior major (RCPma).
3. Obliquus capitis inferior (OCI).
4. Nuchal ligament.

This system is characterized by bundles of fibers arranged parallel to each other in a longitudinal manner. It is assumed that the physiology of the MDB includes sensorimotor functions, postural control, and cerebrospinal fluid (CSF) flow modulation. This occurs by tensioning the SDM or transmitting the force generated by the movements of the head on the SDM itself (Zheng et al., 2018).

It is important to underline the relationship between the cranial sphere and meningeal fascia. Communication points between the pericranial musculature and the SDM are present, allowing muscles to modulate dural tensional states and vice versa. This hypothesis is supported by the presence of esocranical trigeminal nerve endings, which innervate the skull myofascial system (Bordoni et al., 2019a). Based on these observations, a sort of “connective pillar” is present and is represented by the spine and all the muscular, fascial, and connective elements which surround it. The inherent stiffness of these tissues should grant stability and functionality to the upright posture and support to the main anti-gravity systems.

**Figure 1:**
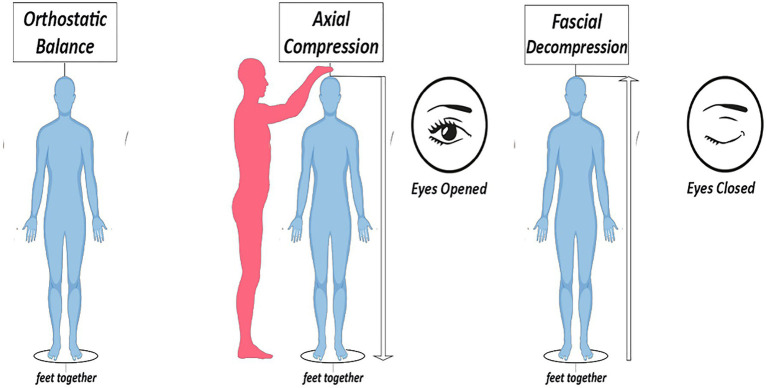
Description of the Pressure and Oscillation Test (POT).

The assessment of anti-gravity and postural function of fasciae should be evaluated through a diagnostic test called Pressure and Oscillation Test (POT), which is subdivided into two phases. The first phase consists of engaging the fascial anti-gravity function through a longitudinal pressure, applied by an operator on a subject from the vertex perpendicular to the ground. By absorbing and transmitting the lines of mechanical stress induced by the operator, the fascial systems accumulate potential energy waiting to be released (the same mechanical principle applicable to springs). By reducing the BoS of the subject, with feet together, a condition of instability is brought about in maintaining the balance of the body mass, stabilized temporarily by the caudal force applied by the operator. In the second phase, the operator rapidly stops the applied force by putting his hand out of the vertex of the subject. In this way, potential energy is suddenly released by the fascial and muscular tissues, in conjunction with closure of the eyes of the subject (Figure 1).

Changes in specific sensorial information, such as closure of the eyes, induce modulations of the whole-body postural oscillations system (Forbes et al., 2018). While the central nervous system is acting to settle a correct neuro-sensorial setting, which is necessary for maintaining an upright posture function, the body mass is under the influence of the force of gravity and passive tissues mechanics, which are disconnected from the central nervous system. At this exact moment, a facilitated oscillation toward the zone of main tissue tensions should emerge.

Considering the major postural instability induced by the reduction of the BoS, before the central nervous system is able to compensate for this condition through the correct activation of the neuromuscular system, it is possible to observe and assess the main oscillation emerging in that specific moment.

An increase of facilitation in swaying toward a specific region of the space inside the COE of an individual should reveal the presence of more stiffness and loss of elasticity in fascial tissues. This would lead the therapist to assess the anatomical region, using first diagnostic and therapeutic approach.

## Discussion

Following this rationale, we were able to build a diagnostic model by determining dysfunctional oscillations patterns, using clinical and the histological (tissues mechanical) evidence behind the POT. A first dysfunctional indicator can be represented by a predominant posterior oscillation on a sagittal plane. This should bring the therapist to further assess the postural/muscle-skeletal/structural system, with the majority of the somatic components relative to this system located behind the “central pillar” of the human body, represented by the spine.

It was clinically observed that a major oscillation occurring in a frontal plane corresponds to alterations of palpable features relative to the meningeal system and its related fascial tissues. The evidence based on stabilometric data observations has shown how subjects affected by vestibular pathologies present increased variations of the center of pressure in a frontal plane compared with a control group (Nacci et al., 2011). The relationship between lateral oscillation and the craniosacral system is also described in the Sacro Occipital Technique (SOT) category II: “Category II involves over-motion or instability of the sacroiliac joint causing a dysfunctional relationship between the tailbone and pelvis. The sacroiliac weight-bearing joint sprain makes it difficult for the body to maintain itself against gravity and function at its optimum. It is common with this category to find low back imbalance, which can affect the knees, shoulders, neck and TMJ with Category II patients” (De Jarnette, 1984). One of the indicators of “category II” (sacroiliac weight-bearing dysfunction) described by De Jarnette (1984) is the lateral sway of the entire spine either to the left or right, away from the center as seen on plumb line analysis (Hochman, 2005). This relationship allows researchers to relate the lateral oscillations on the frontal plane with the craniosacral system and, therefore, with the meningeal system. Further studies and insights in this regard are necessary.

On the other hand, anterior oscillations on a sagittal plane are characterized by changes in the structures of the visceral fascial systems classified as separating fascia 17, and are anatomically distributed behind the spine (Kumka and Bonar, 2012). The mechanics of visceral fascial tissues reflect the functionality analyzed so far to be typical of connective cells, with an extracellular matrix rich in elastin and parallelly arranged collagen bands capable of giving shape and structure to the internal organs and ensuring the transmission of forces. The exact type of innervation of many visceral bands is still uncertain. However, it is known that the parietal peritoneum has the same type of somatic innervation typical of the abdominal wall (Stecco et al., 2017).

**Figure 2:**
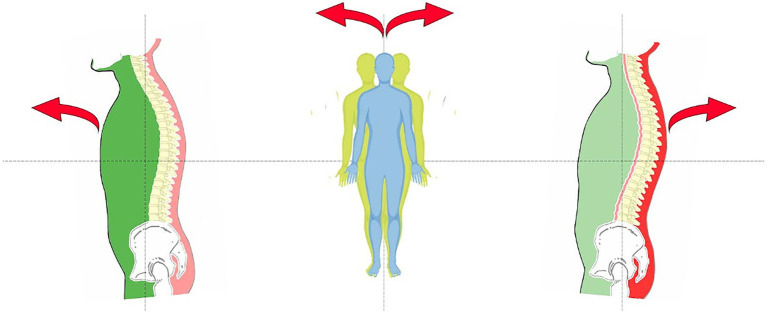
Visceral Pattern (Green) – Meningeal/Cranial Pattern (Yellow) – Postural/muscle skeletal pattern (Red).

Two different types of fascial tissue cover and give structure to each organ. A thin layer intimately adhering to each organ represents the first one, called investing fascia. It probably has an autonomic innervation and a prevalent elastic component. The second type, called insertional fascia, is thicker, fibrous, and multi-layered and functionally connects the various organs to the abdominal walls and to the musculoskeletal system. Between the various layers, there is a loose connective tissue that allows free sliding and motility to the respective organs. Therefore, alterations in tensional forces acting on visceral fascial tissues, through dysfunction in somatic components or an increase in tensions due to autonomic non-striated muscle cells, should act on and transmit directly to the postural somatic system (Bordoni et al., 2019b; Figure 2).

The evidence based on the physiology of the fascial tissues and on naturally expressed unstable balance through Newtonian mechanics support the clinical rationale behind the POT. A first evaluation and measurement method is underway to confirm the clinical validity inherent to the POT. This is done using a randomized clinical protocol that compares the biomechanical analyses of the COE of a group of subjects with respective manual tests of pressure and oscillations. A statistically significant outcome between the POT and the mechanical evaluation method would give substantial validity to the ability of the diagnostic test to express and broadly interpret the COE of a single individual. Future research should add a control group to compare a specific biomechanical analysis of the COE with oscillations observable exclusively through the closed eyes of the subjects.

## Data Availability Statement

The original contributions presented in the study are included in the article/supplementary material, further inquiries can be directed to the corresponding author.

## Author Contributions

AB, SC, GF, JP, SM, PA, and AR contributed to the conception and design of the study. NM, DD, and MC were responsible for drafting and finalizing the manuscript. All authors contributed to the article and approved the submitted version.

### Conflict of Interest

The authors declare that the research was conducted in the absence of any commercial or financial relationships that could be construed as a potential conflict of interest.
